# Impact of CYP3A5 1* and 3* single nucleotide variants on tacrolimus pharmacokinetics and graft rejection risk in pediatric kidney transplant patients

**DOI:** 10.3389/fphar.2025.1592134

**Published:** 2025-05-13

**Authors:** Lesly Yanira Xajil-Ramos, Jesús Alonso Gándara-Mireles, Rodrigo José Vargas Rosales, Oscar Kevin Sánchez García, Andrea Mariela Ruano Toledo, Amy Kateleen Aldana de la Cruz, Ismael Lares-Asseff, Leslie Patrón-Romero, Horacio Almanza-Reyes, Randall Lou-Meda

**Affiliations:** ^1^ Pharmacogenetics and Pharmacogenomics Research Unit, Faculty of Chemical Sciences and Pharmacy, University of San Carlos de Guatemala, Guatemala, Guatemala; ^2^ Pediatric Kidney Disease Research Center at FUNDANIER, Guatemala, Guatemala; ^3^ Latin American Network for the Implementation and Validation of Pharmacogenomics Clinical Guidelines (RELIVAF-CYTED), Santiago, Chile; ^4^ Doctorate Program in Biomedical Sciences, Faculty of Medical Sciences, University of San Carlos de Guatemala, Guatemala, Guatemala; ^5^ Department of Genomics, Interdisciplinary Research Center for Regional Comprehensive Development Durango Unit, National Polytechnic Institute (IPN), Durango, Mexico; ^6^ Galileo University, Guatemala, Guatemala; ^7^ Latin American Society for Pharmacogenomics and Personalized Medicine (SOLFAGEM), Santiago, Chile; ^8^ Faculty of Medicine and Psychology of the Autonomous University of Baja California, Tijuana, Mexico

**Keywords:** CYP3A5, kidney transplant recipients, pharmacokinetics, tacrolimus, pharmacogenetics, acute rejection, individualized dosing

## Abstract

Tacrolimus, a calcineurin inhibitor, is widely used to prevent allograft rejection in kidney transplant recipients. Its metabolism is predominantly mediated by the cytochrome P450 3A5 (CYP3A5) enzyme, and single nucleotide variants (SNVs) within intron 3 of the *CYP3A5* gene are strongly associated with interindividual variability in enzyme expression and activity. These SNVs can generate a cryptic splice site, resulting in either preserved enzymatic function classified as expressers (*CYP3A5* *1/*1 and *1/*3) or loss of function, classified as non-expressers (*CYP3A5* *3/*3). Differential expression of *CYP3A5* contributes to variability in tacrolimus pharmacokinetics and clinical outcomes, including graft rejection and therapeutic efficacy. In this study, we evaluated three pharmacokinetic parameters: trough concentration (TAC-C_0_), weight-adjusted daily dose (TAC-D, mg/kg), and dose-normalized trough concentration (TAC-C_0_/D). One-way ANOVA was used to assess differences in these parameters between *CYP3A5* expressers and non-expressers. Additionally, Poisson regression was performed to examine associations between clinical/genetic variables and the incidence rate of acute rejection events. Genotyping was conducted in 45 pediatric kidney transplant recipients. The *CYP3A5* *3/*3 genotype was most prevalent (66.7%), followed by *1/*3 (26.7%) and *1/*1 (6.7%). During the 6-month post-transplant period, *CYP3A5* expressers required significantly higher tacrolimus doses to achieve target trough levels. Increased drug exposure was associated with a higher incidence of rejection events, whereas *CYP3A5* expression correlated with a reduced rate of rejection. These findings underscore the clinical utility of *CYP3A5* genotyping for optimizing tacrolimus dosing strategies. Carriers of functional *CYP3A5* alleles (*1/*3 or *1/*1) benefit from individualized dose adjustments to achieve therapeutic concentrations and reduce the risk of graft rejection.

## Graphical Abstract

**Figure:**
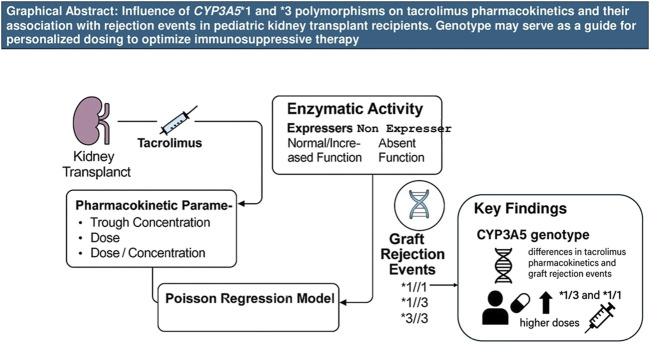
Influence of CYP3A5*1 and *3 variants on tacrolimus pharmacokinetics and their association with rejection events in pediatric kidney transplant recipients. Genotype may serve as a guide for personalized dosing to optimize immunosuppressive therapy.

## Introduction

Kidney transplantation is the preferred treatment for pediatric patients with end-stage kidney disease (ESKD), and the success of the procedure is heavily dependent on lifelong immunosuppressive therapy to ensure transplanted organ survival (Htun et al., 2020). Tacrolimus (TAC), a calcineurin inhibitor, is a cornerstone of immunosuppressive regimens; however, it is characterized by a narrow therapeutic index and considerable inter- and intra-individual pharmacokinetic variability. Consequently, regular monitoring of blood tacrolimus concentrations is essential to maintain therapeutic levels, balance immunosuppressive efficacy, minimize the risk of toxicity, and ensure long-term transplanted organ viability (Degraeve et al., 2020). Tacrolimus pharmacokinetics is influenced by numerous clinical and genetic factors, including ethnicity, renal function, and genetic polymorphisms in drug-metabolizing enzymes (Krall et al., 2021). TAC is primarily metabolized by cytochrome P450 enzymes, notably CYP3A4 and CYP3A5. Among these, CYP3A5 activity is modulated by single nucleotide variants (SNVs) in intron three of the *CYP3A5* gene. These SNVs can generate a cryptic splice site that disrupts normal mRNA splicing, resulting in either active enzyme expression seen in carriers of the *1 allele (*CYP3A5* *1/*1 or *1/*3, “expressers”) or a truncated, non-functional enzyme in homozygous *3 allele carriers (*CYP3A5* *3/*3, “non-expressers”) (Yu et al., 2018). Multiple studies have demonstrated that CYP3A5 expressers exhibit significantly lower plasma TAC concentrations when administered standard doses, placing them at increased risk of acute rejection due to subtherapeutic exposure. Conversely, non-expressers are more likely to develop TAC-associated nephrotoxicity owing to elevated systemic drug levels (Yu et al., 2018; Cheung et al., 2020). The prevalence of the *CYP3A5* *3 allele varies substantially across populations, with notable differences between global and Latin American cohorts (Reséndiz-Galván et al., 2019). This interpopulation variability emphasizes the clinical relevance of characterizing *CYP3A5* genotype frequencies within specific demographic groups and assessing their implications for tacrolimus dosing strategies and clinical outcomes. Genotype-guided immunosuppressive therapy, particularly involving the *CYP3A5* polymorphism, has emerged as a promising approach to optimizing TAC therapy. Integrating pharmacogenetic insights into clinical practice may help reduce the incidence of rejection and toxicity by facilitating individualized dosing protocols (Birdwell et al., 2015). Previous studies have examined the impact of the *CYP3A5* genotype on tacrolimus pharmacokinetics and treatment outcomes, showing that daily dose requirements differ based on genotype (Pasari et al., 2022), recipient ethnicity (Galaviz-Hernández et al., 2020), and post-transplant time intervals (Yanik et al., 2019). Furthermore, CYP3A5 expression status has been implicated in influencing the rate of acute graft rejection (Tang et al., 2011). Given the documented influence of *CYP3A5* polymorphisms on tacrolimus metabolism and the known variability of allele distribution across populations, this study aimed to determine the allelic frequencies of *CYP3A5* variants in pediatric kidney transplant recipients in Guatemala and to evaluate their association with tacrolimus pharmacokinetics and clinical outcomes.

## Materials and methods

A descriptive, retrospective study was conducted involving all pediatric kidney transplant recipients under active follow-up at the Nephrology, Hypertension, Dialysis, and Transplant Service of FUNDANIER, Department of Pediatrics, Roosevelt Hospital, Guatemala City, from 2010 to 2023. The study was conducted in accordance with the ethical principles outlined in the Declaration of Helsinki and received approval from the Independent Ethics Committee of Roosevelt Hospital (Approval No. 179–2023). Written informed consent and assent were obtained from all participants and their legal guardians. Patients were stratified into two groups based on the presence of specific single nucleotide variants (SNVs) in the *CYP3A5* gene: expressers, carrying at least one *CYP3A5* *1 allele (*1/*1 or *1/*3), and non-expressers, homozygous for the *3 allele (*3/*3). Expressers were presumed to have increased CYP3A5 enzymatic activity, leading to faster tacrolimus metabolism, whereas non-expressers were considered to have absent or significantly reduced enzymatic activity, resulting in slower drug clearance.

### Genotyping of CYP3A5 variants

Genomic DNA was extracted from peripheral blood using the ReliaPrep™ gDNA Tissue Miniprep System (Promega), according to the manufacturer’s protocol. Genotyping for *CYP3A5* *1 and *3 alleles was performed using restriction fragment length polymorphism (RFLP) analysis, as previously described (Muller et al., 2020). Polymerase chain reaction (PCR) amplification was carried out in a final reaction volume of 25 µL, comprising 12.5 µL of GoTaq^®^ Hot Start Colorless Master Mix (Promega), 0.6 µL of each primer (10 µM; Inqaba Biotechnical Industries, SA), 1.2 µL of genomic DNA (<250 ng), and nuclease-free water. The PCR products were digested with the SspI restriction enzyme (Thermo Fisher Scientific) by incubation at 36°C for 16 h. Genotypes were determined based on the pattern of DNA fragments observed via agarose gel electrophoresis: Three fragments of 168, 148 and 125 bp for genotype *1/*3, two fragments of 168 and 125 bp for genotype *3/*3 and two fragments of 148 and 125 bp for the *1/*1 genotype (Muller et al., 2020).

### Tacrolimus pharmacokinetic monitoring

Tacrolimus trough concentrations (C_0_) were measured 6 months post-transplantation using 3 mL of peripheral blood collected in EDTA tubes. Quantification was performed using the Alinity i TAC assay (Abbott Laboratories, 2019), a chemiluminescent microparticle immunoassay (CMIA) that utilizes anti-TAC antibody-coated microparticles and an acridinium-labeled TAC conjugate. The resulting luminescent signal was quantified in relative light units (RLU) to determine TAC concentration. The following pharmacokinetic parameters were analyzed: Trough concentration (TAC-C_0_): Plasma concentration at time zero, immediately before administration of the oral dose (6 months after kidney transplantation). Weight-normalized daily dose (TAC-D): Calculated as the daily oral TAC dose divided by body weight (mg/kg) (6 months post-transplant). Dose-normalized trough concentration (TAC-C_0_/D): Calculated by dividing TAC-C_0_ by TAC-D (ng/mL per mg/kg). The therapeutic target for plasma TAC concentration 6 months post-transplant was defined as 7–10 ng/mL.

### Evaluation of acute graft rejection

Acute graft rejection was assessed by calculating the Event Rate, defined as the number of clinically confirmed rejection episodes per patient within the first 6 months post-transplantation. Events were identified retrospectively through review of electronic medical records. Diagnosis of acute rejection was made by the attending nephrologist based on established institutional criteria, including persistent elevation of serum creatinine and urea, decreased urine output and histopathological confirmation via renal biopsy consistent with acute rejection. These criteria reflect the standard diagnostic practices used at the FUNDANIER transplant unit at Roosevelt Hospital.

### Statistical analysis

To assess the population distribution of *CYP3A5* alleles and genotypes, a Pearson chi-square test was performed, and Hardy-Weinberg equilibrium (HWE) was evaluated. Descriptive analyses of pharmacokinetic parameters included the generation of box plots to illustrate the median and interquartile ranges for trough concentration (TAC-C_0_), weight-normalized daily dose (TAC-D, mg/kg), and dose-normalized trough concentration (TAC-C_0_/D). Given that body weight significantly influences tacrolimus pharmacokinetics particularly in pediatric populations normalization procedures were implemented. Specifically, the daily tacrolimus dose was adjusted for patient weight (TAC-D), and the trough concentration was expressed relative to the weight-adjusted dose (TAC-C_0_/D). These transformations enabled more accurate interindividual comparisons by minimizing variability attributable to body size and isolating the effect of *CYP3A5* genetic variants on drug exposure. Normality and homogeneity of variance were assessed prior to hypothesis testing. All dependent variables (TAC-C_0_, TAC-D, and TAC-C_0_/D) were log-transformed to approximate normal distributions. The Shapiro-Wilk test was used to confirm the normality of residuals, while Levene’s test evaluated the homogeneity of variances across groups. Following confirmation of these assumptions, a one-way analysis of variance (ANOVA) was conducted to identify statistically significant differences in pharmacokinetic parameters between expressers (*CYP3A5* *1/*1 and *1/*3) and non-expressers (*CYP3A5* *3/*3). All statistical analyses were performed using R software (version 4.4.1), and a two-sided p-value of <0.05 was considered statistically significant. A complementary genetic association analysis was conducted to evaluate the relationship between *CYP3A5* genotype and acute graft rejection. A Poisson regression model was applied to assess the influence of tacrolimus pharmacokinetics (TAC dose and plasma concentration) and genotype on rejection event rates. Incidence Rate Ratios (IRRs) were calculated to estimate the magnitude of association for each variable. To further explore the impact of genotype on clinical outcomes, inheritance models were applied, including dominant (*1/*1 + *1/*3 vs. *3/*3), recessive (*1/*1 vs. *1/*3 + *3/*3), and additive (based on the number of functional *1 alleles, ranging from 0 to 2) were applied. For each model, odds ratios (ORs) and 95% confidence intervals (CIs) were calculated using logistic regression. Statistical significance was defined as p < 0.05, and results were interpreted within the context of the observed clinical outcomes, particularly the incidence of acute rejection.

## Results

A total of 45 pediatric kidney transplant recipients of native Guatemalan origin were included in this retrospective study. All patients met the predefined eligibility criteria and were under active follow-up at the time of analysis. Due to the observational nature of the study, no *a priori* sample size calculation was performed; instead, the entire eligible cohort during the 2010–2025 period was included to maximize representativeness. To assess the statistical robustness of the findings, *post hoc* power analyses were conducted, offering insight into the strength of the observed associations.

Table 1 presents the demographic, clinical, and biochemical characteristics of the cohort. The leading cause of end-stage kidney disease (ESKD) was kidney disease of unknown etiology. The vast majority of transplants (90.5%) were performed with living-related donors. At the time of transplantation, patient age ranged from 4.1 to 20.3 years. Induction immunosuppression consisted of basiliximab for recipients of living-donor grafts and thymoglobulin for recipients of deceased-donor grafts, in combination with corticosteroids. Maintenance immunosuppressive therapy included steroids, mycophenolate mofetil or azathioprine, and tacrolimus, initiated at 0.1–0.3 mg/kg/dose administered orally every 12 h, with adjustments based on therapeutic drug monitoring.

**TABLE 1:** Demographic, clinical and laboratory characteristics of pediatric kidney transplant recipients.

Characteristics	Patients (N = 45)
Age*, at time of transplantation (years) Range	13.4 ± 3.9 (4.1–20.3)
Sex, Male/Female	27/18
Weight* at time of transplantation (kg)	43.7 ± 15.4
Height* at time of transplantation (cm)	144.7 ± 14.1
BMI (kg/m2)*	20.5 ± 5.3
Cause of ESKD	
Kidney Disease of Unknown Etiology	27 (60.0%)
Vesicoureteral reflux	8 (17.8%)
Glomerulonephritis	2 (4.4%)
Others	8 (17.8%)
Type of donor	
Living related/unrelated	38 (84.4%)/3(6.7%)
Cadaveric related/unrelated	0 (0%)/4 (8.9%)
Immunosuppressant use	
Prednisone	45 (100%)
Mycophenolate	33(73.3%)
Azathioprine	12 (26.7%)
Tacrolimus dose in mg/day*	4.6 ± 2.7
Tacrolimus level in ng/mL*	7.7 ± 4.9
Serum creatinine, mean ± SD at 6 months post-transplant (mg/dL)	1.8 ± 1.1
Serum albumin, mean ± SD at 6 months post-transplant (g/dL)	4.8 ± 0.3
Serum Urea, mean ± SD at 6 months post-transplant (mg/dL)	24.3 ± 13.1
Hematocrit, mean ± SD at 6 months post-transplant (%)	37.3 ± 4.7

ESKD: End-stage kidney disease; *Mean ± SD; N: total number patients.

Table 2 summarizes the allelic, genotypic, and phenotypic frequencies of the *CYP3A5* SNVs *1 and *3 (rs776746). The most frequent genotype was *3/*3 (66.6%), followed by *1/*3 (26.6%), and *1/*1 (6.6%). Consequently, a lower proportion of patients were classified as *CYP3A5* expressers (*1/*1 or *1/*3), while the majority were non-expressers (*3/*3), indicating a predominance of reduced CYP3A5 enzymatic activity in this cohort.

**TABLE 2:** Allelic, genotypic and phenotypic frequencies of single nucleotide variants of CYP3A5 (rs776746) in patients treated with tacrolimus.

CYP3A5 rs776746	N = 45
Genotypes	n (%)
*1/*1	3 (6.7)
*1/*3	12 (26.7)
*3/*3	30 (66.7)
Alleles	
*1	18 (20.0)
*3	72 (80.0)
Phenotypes	
Expressor	15 (33.3)
Non Expressor	30 (66.7)

N: Total number patients; n (%): Frecuency.

**FIGURE 1:**
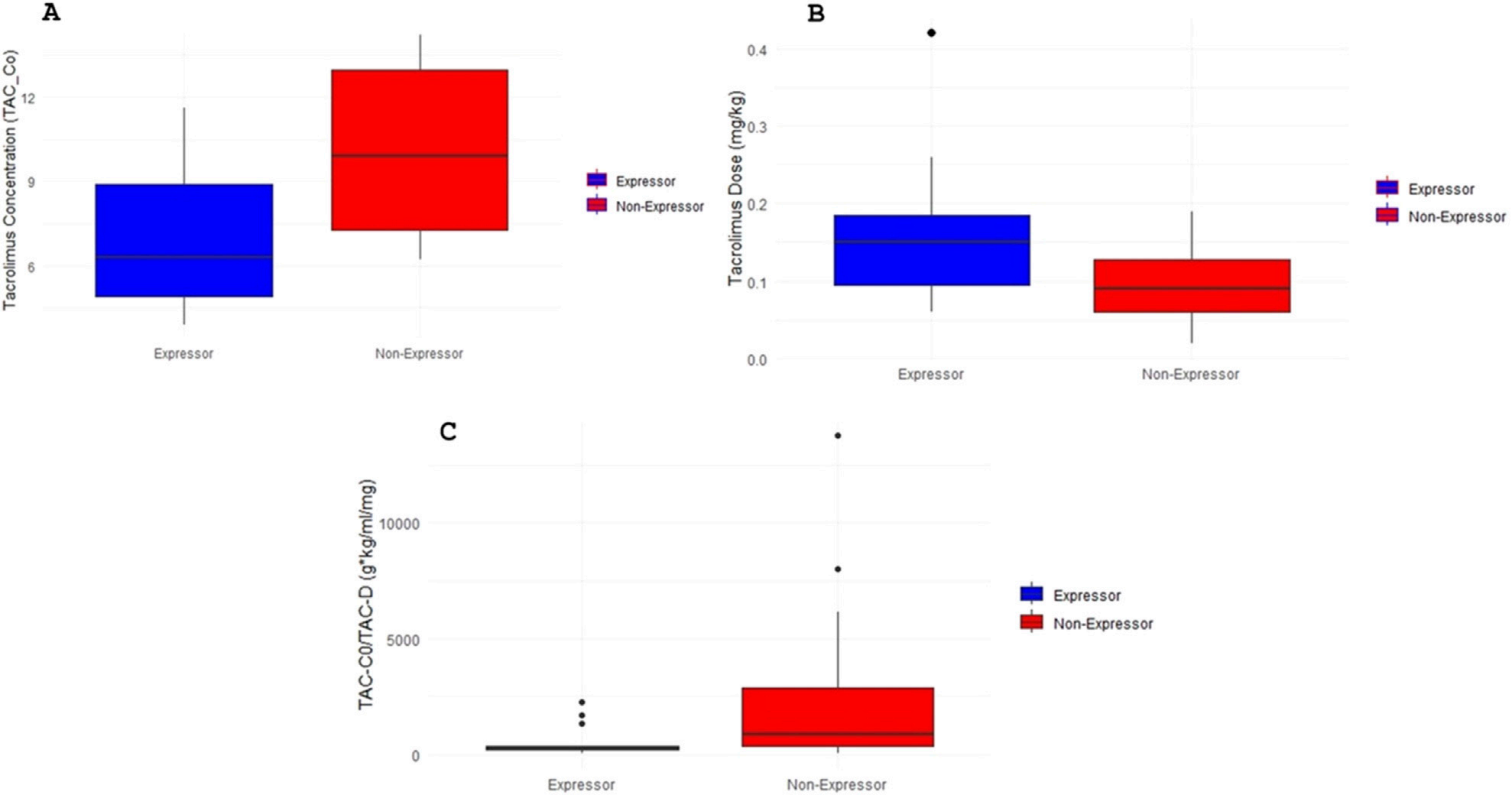
Tacrolimus pharmacokinetics according to CYP3A5 genotype. **(A)** Plasma trough tacrolimus levels (TAC-C0 ng/ml) based on CYP3A5 genotype of the patients included in the study. The box plot represents plasma trough tacrolimus levels data grouped into CYP3A5 expressers and non-expressers patients. **(B)** Daily tacrolimus dose requirements normalized by weight (TAC-D mg/kg) according to CYP3A5 genotype. The box plot represents data grouped into CYP3A5 expressers and non-expressers patients. **(C)** Plasma trough tacrolimus levels normalized to daily dose requirement (TAC-C0/TAC-D g*kg/ml/mg) according to CYP3A5 genotype. The box plot represents data grouped into CYP3A5 expressers and non-expressers patients.

Figure 1A shows the comparison of plasma tacrolimus trough concentrations (TAC-C_0_) at 6 months post-transplant between expressers and non-expressers. No statistically significant difference was observed between groups (p > 0.05). In contrast, Figure 1B illustrates a significantly higher weight-normalized daily dose requirement (TAC-D, mg/kg) among expressers to reach target plasma concentrations, as confirmed by ANOVA (p < 0.05). Figure 1C displays the dose-normalized trough concentrations (TAC-C_0_/D), demonstrating significantly lower drug exposure in expressers compared to non-expressers (p < 0.05), consistent with increased metabolic clearance in the presence of functional CYP3A5 enzyme.

**FIGURE 2:**
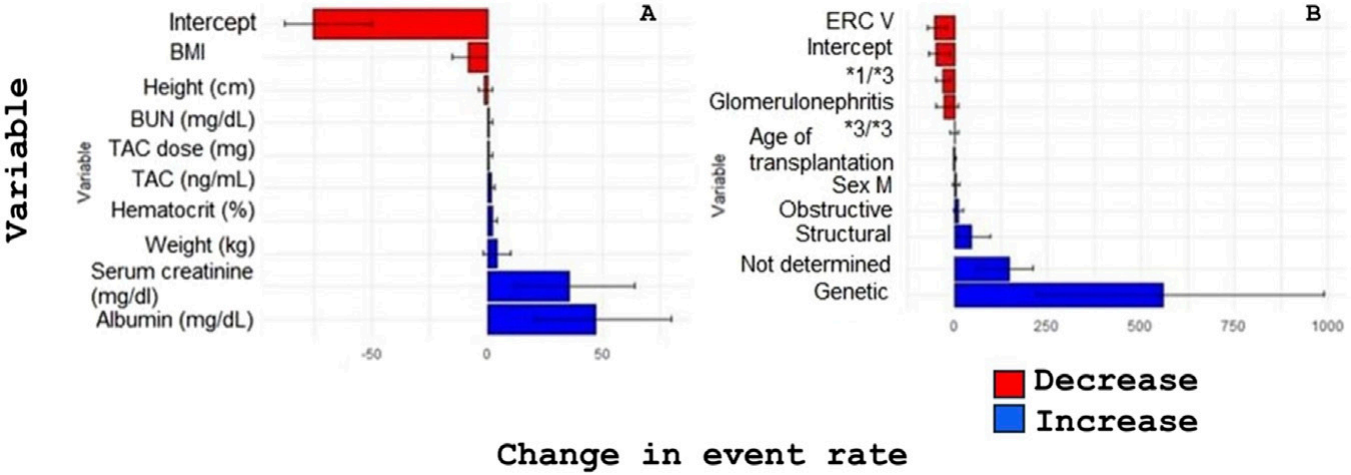
Effect of clinical and genetic factors on rejection rates and percentage change in graft rejection. **(A)** Impact of variables on the rate of rejection events of the grafted organ. **(B)** Impact of variables on the percentage change of rejection events of the grafted organ.

To assess clinical outcomes, a Poisson regression analysis was conducted to examine the relationship between tacrolimus pharmacokinetics, CYP3A5 genotype, and graft rejection events (Table 3). Significant associations were found between both TAC dose and plasma concentration and the incidence rate of acute rejection (p < 0.05). Specifically, each 1 mg increase in tacrolimus dose was associated with a 0.94% increase in the rejection event rate (IRR = 1.0094), while each 1 ng/mL increase in TAC concentration correlated with a 1.77% increase in event rate (IRR = 1.0177). These findings underscore the importance of dose optimization and therapeutic drug monitoring in minimizing adverse outcomes. Furthermore, patients with functional *CYP3A5* alleles (*1/*3 or *1/*1) demonstrated lower rates of acute rejection compared to non-expressers, suggesting a potential protective effect associated with active enzyme expression (Figures 2A,B).

**TABLE 3:** Analysis of clinical and genetic variables associated with the event rate in kidney transplant patients.

Variable	IRR	% Change in event rate	IC 95%	p-value
First model				
Intercept	0.2443	−75.57%	[0.12, 0.50]	<0.001
Weight (Kg)	1.0406	+4.06%	[0.98, 1.10]	0.10
Height (Cm)	0.9883	−1.17%	[0.96, 1.02]	0.32
BMI (kg/m2)*	0.9190	−8.10%	[0.85, 1.00]	0.05
TAC Dose (mg)	1.0094	+0.94%	[1.00, 1.02]	0.04
TAC concentration [ng/mL]	1.0177	+1.77%	[1.01, 1.03]	0.01
Serum creatinine (mg/dL)	1.3559	+35.59%	[1.12, 1.64]	<0.01
Blood Urea Nitrogen (mg/dL)	1.0077	+0.77%	[1.00, 1.02]	0.03
Albúmin (mg/dL)	1.4697	+46.97%	[1.20, 1.80]	<0.001
Hematocrit (%)	1.0230	+2.30%	[1.00, 1.04]	0.04

*Body mass index; IRR: incidence rate ratio; IC 95%: 95% confidence interval; p-value: Statistic significance.

A complementary genetic association analysis was performed to explore the risk of graft rejection across different *CYP3A5* inheritance models (Table 4). In the dominant model (*1/*1 + *1/*3 vs. *3/*3), carriers of the *1 allele exhibited a trend toward reduced risk (OR = 0.47; 95% CI: 0.13–1.64), though not statistically significant. In the recessive model (*1/*1 vs. *1/*3 + *3/*3), the observed OR was 1.90 (95% CI: 1.16–21.88), suggesting a possible increased risk among homozygous expressers, though this may be influenced by the small number of *1/*1 individuals. The additive model, which assessed the effect of increasing numbers of functional *1 alleles, yielded an OR of 0.75 (95% CI: 0.29–1.07), reflecting a non-significant protective trend per additional *1 allele. These findings highlight the relevance of *CYP3A5* genotype in tacrolimus pharmacokinetics and its potential impact on graft rejection risk, reinforcing the utility of pharmacogenetic-guided therapy in pediatric kidney transplantation.

**TABLE 4:** Association between CYP3A5 genotype and risk of graft rejection under different inheritance models.

Inheritance model	Comparison	Rejection (N)	No rejection (N)	Odds ratio (IC 95%)	p-value	Interpretation
Dominant	*1/*1 +*1/*3 vs. *3/*3	6 vs. 18	9 vs 13	0.47 (0.13–1.04)	0.10	Not significant, possible protective effect
Recessive	*1/*1 vs. *1/*3 +*3/*3	2 vs. 22	1 vs 22	1.90 (1.16–21.88)	0.20	Not significant
Additive	Per number of *1 alleles (0–2)	24	22	0.75 (0.29–0.07)*	0.06	Not significant, protective trend

*Estimated Odds Ratio using additive model; N: total number patients; IC 95%: 95% confidence interval; p-value: Statistic significance.

## Discussion

Calcineurin inhibitors, such as tacrolimus (TAC), remain a cornerstone of immunosuppressive therapy in kidney transplantation. However, their clinical use necessitates continuous therapeutic drug monitoring to maintain target plasma levels, ensuring a balance between efficacy and safety to prolong transplanted organ survival (Pasari et al., 2022; Bezerra et al., 2020). Interindividual variability in TAC pharmacokinetics particularly in pediatric populations necessitates individualized dosing strategies. Genetic polymorphisms, notably in drug-metabolizing enzymes such as CYP3A5, are increasingly recognized as critical determinants of interpatient variability in TAC exposure (Buendía et al., 2020).

In the current study, we investigated the influence of *CYP3A5* genotype on TAC pharmacokinetics in a cohort of Guatemalan pediatric kidney transplant recipients. This is of clinical importance, as systemic exposure to TAC directly affects both the immunosuppressive efficacy and toxicity profile of the drug. Despite current dosing adjustments being performed empirically in this population, our findings underscore the value of pharmacokinetic monitoring and support the implementation of genotype-guided dosing protocols to optimize treatment outcomes (Vecchia Genvigir et al., 2019). Consistent with previous reports in Latin American populations, the *CYP3A5* *3/*3 genotype associated with loss of enzyme function was the most frequent variant observed in our cohort (Table 2). This is in line with prior studies in mestizo populations, which reflect historical admixture from European and African ancestries and its influence on the genetic architecture of the Guatemalan Mayan population (Krall et al., 2021; Reséndiz-Galván et al., 2019; Galaviz-Hernández et al., 2020; Muller et al., 2020; Alatorre-Moreno et al., 2024; Hannachi et al., 2021; Cusinato et al., 2014).

Figure 1A illustrates the comparison of trough plasma tacrolimus concentrations (TAC-C_0_) at 6 months post-kidney transplantation, stratified by *CYP3A5* expression genotypes. Although mean concentrations differed between genotypic groups, analysis of variance (ANOVA) did not reveal statistically significant differences (p > 0.05). To more accurately assess the pharmacokinetic exposure to tacrolimus, additional variables were analyzed at the same time point. In Figure 1B, weight-normalized daily tacrolimus dose requirements (TAC-D, mg/kg) are compared across *CYP3A5* expression genotypes. Figure 1C presents the dose-adjusted trough concentrations (TAC-C_0_/TAC-D), which reflect overall drug exposure. In both cases, ANOVA revealed statistically significant differences between expressers (*CYP3A5* *1/*1 and *1/*3) and non-expressers (*3/*3) (p < 0.05). Specifically, patients classified as non-expressers (*CYP3A5* *3/*3) required approximately 50% lower daily doses (mg/kg/day) than those categorized as expressers to achieve comparable trough concentrations. These results suggest that the absence of functional *CYP3A5* enzyme activity is associated with reduced drug clearance and higher systemic exposure, consistent with a well-established pharmacogenetic influence on tacrolimus pharmacokinetics. This genotype-dependent variability has been previously documented in several studies (Yanik et al., 2019; Vecchia Genvigir et al., 2019; Hannachi et al., 2021; Hu et al., 2018). For instance, a study conducted in Chilean pediatric transplant recipients demonstrated significant differences in tacrolimus exposure, including the area under the concentration–time curve (AUC) calculated using an abbreviated four-point model (0, 1, 2, and 4 h post-dose), according to *CYP3A5* genotype (Krall et al., 2021).

Although pharmacogenetic studies in Latin American populations remain limited, research conducted in Mexican kidney transplant recipients similarly showed that *CYP3A5* genotype is strongly associated with tacrolimus pharmacokinetic variability. At 1 month post-transplant, carriers of functional alleles had up to a fourfold increased probability of exhibiting TAC concentrations >15 ng/mL, depending on genotype. Moreover, a strong interaction with concomitant medications was observed, with a ninefold increased risk of supratherapeutic TAC levels in patients receiving agents known to elevate tacrolimus plasma concentrations (Alatorre-Moreno et al., 2024). Together, these findings provide compelling external validation for the results observed in the present study and reinforce the clinical relevance of *CYP3A5* genotype as a determinant of tacrolimus dose requirements and exposure.

Previous studies have demonstrated that the area under the concentration–time curve (AUC) is a robust pharmacokinetic indicator for evaluating the association between therapeutic response and *CYP3A5* genotype. Patients with the *CYP3A5* *3/*3 genotype have been shown to exhibit significantly higher AUC values, adjusted for weight-normalized daily dose, compared to expressers. Specifically, AUC values were reported to be 1.56-fold higher than those in *1/*3 carriers and 1.91-fold higher than in *1/*1 carriers. These differences remained consistent across various post-transplant phases during longitudinal therapeutic monitoring, underscoring the sustained impact of *CYP3A5* polymorphisms on tacrolimus exposure over time (Krall et al., 2021). Consistent with these findings, our results (Figure 1B) showed that patients with *CYP3A5* expressor genotypes (*1/*1 and *1/*3) required significantly higher daily doses, normalized by body weight, to attain target plasma concentrations. This observation aligns with the well-established pharmacogenetic mechanism whereby functional CYP3A5 expression leads to increased enzymatic activity, faster tacrolimus clearance, and consequently lower drug exposure at standard doses (Vecchia Genvigir et al., 2019; Chen et al., 2020). These data are supported by the Clinical Pharmacogenetics Implementation Consortium (CPIC) guidelines, which recommend increasing the starting dose of tacrolimus by 1.5 to 2-fold in patients identified as *CYP3A5* expressers to achieve target therapeutic levels more rapidly (Birdwell et al., 2015). Importantly, *CYP3A5* genotype has also been associated with various clinical outcomes beyond dose requirements, including the time required to reach therapeutic concentrations, risk of graft rejection, and susceptibility to adverse drug reactions (ADRs) such as nephrotoxicity resulting from prolonged TAC overexposure (Cheung et al., 2020; Yanik et al., 2019; Tang et al., 2011; Rath, 2013). In our study, the clinical implications of overexposure were reflected in the analysis of rejection event rates. Both higher TAC doses and elevated plasma concentrations were significantly associated with increased rates of acute graft rejection (p < 0.05). Specifically, for each 1 mg increase in TAC dose, the event rate rose by 0.94% (IRR = 1.0094), while each 1 ng/mL increase in TAC plasma levels was associated with a 1.77% increase in rejection risk (IRR = 1.0177). These results emphasize the critical need for precise therapeutic monitoring and suggest that even modest deviations in TAC exposure can result in clinically meaningful consequences.

Interestingly, patients carrying at least one functional *CYP3A5* allele (*1/*1 or *1/*3) experienced lower rates of rejection compared to those with the *3/*3 genotype, suggesting a protective effect potentially mediated by more predictable and stable TAC pharmacokinetics. This trend was further evaluated using dominant, recessive, and additive inheritance models (Table 4). While the dominant model showed a non-significant trend toward reduced rejection risk in expressers (OR = 0.47; 95% CI: 0.13–1.64), the additive model indicated a moderate, albeit non-significant, protective effect associated with each additional functional allele (OR = 0.75; 95% CI: 0.29–1.07). Conversely, the recessive model yielded an OR > 1 (OR = 1.90; 95% CI: 1.16–21.88), though this estimate was imprecise due to the low frequency of *1/*1 homozygotes in the sample. While none of these associations reached statistical significance, likely due to the limited sample size, they collectively suggest a potential link between the *3/*3 genotype and increased risk of graft rejection, consistent with prior literature (Warzyszyńska et al., 2022).

In this study, the therapeutic response was evaluated based on the incidence of graft rejection events, which aligned with our primary objective of assessing the clinical relevance of *CYP3A5* genotyping in relation to tacrolimus pharmacokinetics and transplant outcomes. Although adverse drug reactions (ADRs) were not specifically assessed, this was a deliberate methodological choice, as the focus was directed toward efficacy-related endpoints. Nevertheless, the role of genotype in predicting TAC-related toxicity remains an important and complementary area of investigation. Future prospective studies incorporating systematic ADR monitoring will be valuable in delineating the dual roles of *CYP3A5* polymorphisms in both therapeutic efficacy and safety outcomes in transplant recipients.

In addition to genetic variables, several studies have examined the influence of clinical and demographic factors on drug safety and efficacy. While this approach has been widely applied in various disease contexts, studies focusing on immunosuppressive therapies in Latin American populations remain limited. Most research in the region has concentrated on oncology. For example, a population pharmacokinetic model developed in Mexican children with acute lymphoblastic leukemia identified covariates such as anti-L-asparaginase antibody presence, sex, and age as influential predictors of pharmacokinetic and pharmacodynamic variability in L-asparaginase therapy. Notably, early asparagine depletion within 24 h was associated with an elevated risk of pancreatitis, suggesting its potential as a predictive biomarker (Gándara-Mireles et al., 2024a). Similarly, a population pharmacokinetic analysis of doxorubicin in Mexican pediatric patients identified genetic polymorphisms and nutritional status, particularly body mass index (BMI), as important determinants of drug clearance and cardiotoxicity risk. Altered pharmacokinetics were observed in patients with low BMI, younger age, or specific genetic variants, further highlighting the multifactorial nature of drug response (Gándara-Mireles et al., 2024b; Gándara-Mireles et al., 2023). These findings collectively underscore the importance of population pharmacokinetic modeling as a tool for optimizing individualized therapy by integrating both genetic and non-genetic covariates. In our study, although we focused on pharmacogenetic influences on tacrolimus pharmacokinetics, we recognize that incorporating additional clinical covariates could enhance predictive accuracy and improve therapeutic outcomes.

### Strengths of the study

One of the principal strengths of this study is its novelty and regional relevance, as it represents one of the first pharmacogenetic evaluations of *CYP3A5* polymorphisms in pediatric kidney transplant recipients in Guatemala. Given the underrepresentation of Latin American populations in pharmacogenomic research particularly within the field of transplantation—this investigation provides region-specific data of significant clinical value. It offers important insights into interethnic variability in *CYP3A5* allele frequencies and their clinical implications, thereby addressing a critical gap in the global pharmacogenetics literature. Another strength lies in the integration of pharmacokinetics and pharmacogenetics. This study effectively combines key pharmacokinetic parameters (TAC-C_0_, TAC-D, and TAC-C_0_/D) with *CYP3A5* genotypic profiles. The use of weight-normalized and dose-adjusted metrics appropriately accounts for interindividual variability in body size, which is particularly relevant in pediatric populations. This methodological approach allows for a physiologically meaningful comparison across genotypic groups, enhances the translational applicability of the findings, and supports the implementation of genotype-based dosing strategies. A further strength is the application of inheritance models to genetic association analysis. The use of dominant, recessive, and additive models to evaluate the influence of *CYP3A5* genotypes on graft rejection outcomes provides a comprehensive framework for interpreting genotype–phenotype relationships. This multidimensional analysis enables a nuanced understanding of the pharmacogenetic impact on clinical outcomes and reinforces the rationale for individualized immunosuppressive therapy based on metabolizer status. The study also demonstrates strong clinical applicability and institutional implementation. A particularly notable strength is the documented integration of *CYP3A5* genotyping into the institutional protocol for pediatric kidney transplantation. This real-world application highlights the translational impact of the study and aligns with current trends in personalized medicine, especially in resource-limited healthcare settings where tailored approaches can have substantial impact.

In contrast to many pharmacokinetic studies that focus exclusively on drug concentrations, this research includes the evaluation of clinically meaningful outcomes, specifically acute graft rejection events, as a therapeutic efficacy endpoint. The observed correlation between increased tacrolimus exposure (both in terms of dose and plasma concentration) and higher rejection event rates underscores the clinical risks associated with overexposure and supports the need for individualized dose optimization based on metabolizer phenotype. The study also exhibits rigorous statistical methodology. It employs appropriate analytical techniques, including logarithmic transformation of non-normally distributed variables, analysis of variance (ANOVA) for group comparisons, and Poisson regression for modeling event rates. Additionally, the use of *post hoc* power analyses enhances the interpretability and robustness of the findings, compensating for the absence of an *a priori* sample size estimation in this retrospective design. Finally, the study adheres to high ethical and methodological standards. Ethical approval and informed consent procedures were conducted in strict accordance with the Declaration of Helsinki. The methodological rigor is further supported by the use of validated genotyping techniques (PCR-RFLP) and standardized therapeutic drug monitoring assays, which together ensure reproducibility, technical validity, and data reliability.

### Study limitations

One major limitation of this study is the relatively small sample size (n = 45), which may have limited the statistical power to detect subtle yet clinically relevant associations particularly in genotype subgroup analyses. For example, the low frequency of the *CYP3A5* *1/*1 genotype restricted the robustness of comparisons across inheritance models (e.g., dominant versus recessive). Consequently, confidence intervals for certain effect estimates were wide, precluding definitive conclusions. Although a *post hoc* power analysis was conducted to assess statistical adequacy, the findings should be interpreted with caution and ideally validated in larger, multicenter cohorts. Another limitation is the single-center design, which may constrain the generalizability of the results. This study was conducted at a single national reference center in Guatemala, and findings may not be directly applicable to broader pediatric transplant populations. Genetic background, clinical protocols, and healthcare infrastructure may vary substantially across regions. As such, extrapolation to populations outside Latin America—or to adult transplant cohorts—should be approached with caution. Further multicenter studies across diverse ethnic groups are warranted to assess reproducibility and external validity. The retrospective nature of the study introduces additional limitations related to data completeness, standardization, and control of confounding variables. For instance, heterogeneity in clinical documentation, timing of laboratory assessments, and dosing adjustment practices could have introduced bias or reduced the precision of pharmacokinetic evaluations. The study also lacks comprehensive clinical outcome measures. While acute kidney transplant rejection was appropriately used as the primary clinical endpoint, other important outcomes such as long-term graft survival, chronic rejection, and adverse drug reactions (e.g., nephrotoxicity, neurotoxicity, and opportunistic infections) were not evaluated. These broader clinical indicators are essential for fully characterizing the impact of pharmacogenetic variability. Their omission may have restricted the scope of the findings and potentially underestimated the broader genotype-associated risks and benefits. A further limitation is the absence of systematic data on drug**–**drug interactions. Tacrolimus pharmacokinetics is highly susceptible to interactions with commonly co-administered agents, including azole antifungals, calcium channel blockers, and macrolide antibiotics. The lack of detailed documentation on concomitant medications limits the ability to adjust for potential confounders. As a result, some observed associations between genotype and drug exposure may have been influenced by unmeasured pharmacokinetic interactions.

Moreover, the study focused exclusively on CYP3A5 polymorphisms, without assessing other pharmacogenetically relevant genes such as *CYP3A4*, *ABCB1* (P-glycoprotein), and *POR* (cytochrome P450 oxidoreductase). These genes are also known to affect tacrolimus metabolism and disposition. The exclusion of these markers may oversimplify the pharmacogenetic landscape and reduce the explanatory power of the model, particularly in patients with atypical pharmacokinetic or clinical profiles. A critical limitation was the absence of longitudinal pharmacokinetic data at multiple clinically relevant time points (e.g., 1, 3, and 6 months post-transplant). This restricted the ability to assess temporal trends in tacrolimus exposure and genotype–pharmacokinetic relationships, especially during the early post-transplant period, which is marked by heightened immunological risk and dynamic dose adjustments. The lack of serial pharmacokinetic data limits the granularity with which genotype effects can be characterized over time. Although trough tacrolimus concentrations (TAC-C_0_) at 6 months were similar across genotypic groups, this may mask earlier differences in drug exposure. Patients carrying functional *CYP3A5* alleles (*1/*1 or *1/*3) exhibit increased metabolic clearance and often require escalated dosing during the early post-transplant period to avoid subtherapeutic exposure. The absence of traceable data on the frequency, timing, and magnitude of dose modifications limits the ability to attribute final drug levels solely to pharmacogenetic variability, rather than to clinical dose optimization practices. Additionally, while the additive genetic model showed a non-significant trend toward a protective effect with each additional *CYP3A5* *1 allele, the lack of detailed longitudinal dosing data weakens the interpretation of this association. This limitation highlights the need for prospective data collection and the proactive integration of pharmacogenetic testing at treatment initiation, rather than relying on reactive dose modifications based on delayed monitoring. Early genotype-guided dosing could reduce variability in drug exposure, minimize the risk of early underexposure in expressers, and lead to more stable immunosuppressive levels ultimately improving clinical outcomes.

### Future research

To build on the findings of the present study and address its methodological limitations, future research should prioritize the design and execution of prospective multicenter cohort studies. Larger, prospective investigations involving multiple transplant centers across Latin America and other underrepresented regions are essential to validate and generalize the observed associations between *CYP3A5* genotype and tacrolimus pharmacokinetics. Such studies should aim to recruit genetically and demographically diverse populations, ensuring adequate representation of all relevant genotypic subgroups particularly *CYP3A5* *1/*1 carriers to improve statistical power and enable more robust genetic association modeling. A second area of future investigation is the implementation of longitudinal pharmacokinetic monitoring, incorporating serial pharmacokinetic sampling at standardized post-transplant intervals (e.g., days 7, 30, 90, and 180). This approach would provide more granular insights into the temporal dynamics of tacrolimus exposure in relation to genotype, including dose titration kinetics, drug accumulation patterns, and variability in clearance across distinct phases of immunosuppressive therapy. Future research should also focus on the integration of comprehensive pharmacogenetic panels. Expanding beyond *CYP3A5*, future studies should include additional pharmacogenes such as *CYP3A4*, *ABCB1*, *POR*, and *NR1I2* (pregnane X receptor), which collectively influence tacrolimus metabolism, transport, and bioavailability. Incorporating these markers would allow for polygenic risk stratification and the development of more precise genotype-guided dosing algorithms.

Another recommended research direction is the development of population pharmacokinetic (PopPK) models. The application of nonlinear mixed-effects modeling to construct PopPK models specifically tailored to pediatric kidney transplant recipients could significantly enhance individualized dose prediction. These models should integrate genetic, demographic (e.g., age, weight, body mass index), and clinical covariates (e.g., renal function, inflammation, co-medications) to simulate drug exposure under various clinical scenarios and support model-informed precision dosing (MIPD) strategies. Evaluation of clinical outcomes beyond acute rejection is also warranted. Future studies should broaden outcome assessments to include long-term graft survival, chronic rejection, time to therapeutic target range, and the incidence of tacrolimus-associated adverse drug reactions (e.g., nephrotoxicity, neurotoxicity, and infections). These outcomes are critical to comprehensively assess the clinical consequences of pharmacogenetic variability and to quantify the long-term benefits of genotype-guided immunosuppressive therapy. Additionally, future research should address the impact of pharmacotherapeutic interactions and co-medications. Prospective studies must systematically evaluate the influence of concomitant medications on tacrolimus pharmacokinetics. This includes assessing the effects of CYP3A enzyme inhibitors and inducers, which may significantly alter drug exposure independent of genetic background. Moreover, future research should explore pharmacogenetic–pharmacokinetic interaction networks, integrating genetic susceptibility with established drug–drug interaction profiles to provide a more comprehensive understanding of interindividual variability in tacrolimus response.

## Conclusion

This study in 45 Guatemalan pediatric kidney transplant recipients identified significant differences in tacrolimus pharmacokinetics according to CYP3A5 genotype. Carriers of at least one functional allele (*1/*1 or *1/*3) required higher weight-adjusted doses to attain therapeutic drug concentrations, consistent with increased metabolic clearance. Although plasma tacrolimus levels at 6 months post-transplant did not significantly differ across genotypes, expressers benefited from early, genotype-informed dose adjustments guided by therapeutic drug monitoring, potentially mitigating the risk of acute rejection.

Based on these findings, we advocate for the routine implementation of CYP3A5 genotype-guided tacrolimus dosing in pediatric kidney transplant recipients. In alignment with this precision medicine approach, CYP3A5 genotyping has been integrated into pre-transplant clinical protocols at our institution to inform initial dosing strategies according to predicted metabolizer status. This targeted approach aims to reduce interindividual variability in early drug exposure, particularly among rapid metabolizers, and enhance immunosuppressive efficacy.

Given the historical underrepresentation of Latin American populations in pharmacogenetic research and the logistical challenges of integrating genomic tools into clinical workflows in the region (Salas-Hernández et al., 2023), this study provides a critical contribution to the fields of clinical pharmacokinetics and pharmacogenomics. To our knowledge, it represents the first pharmacogenetic evaluation of tacrolimus response in Guatemalan pediatric transplant patients and establishes a foundation for future studies incorporating genetic, clinical, and demographic variables. As seen in oncology, the application of population pharmacokinetic modeling may further advance individualized immunosuppression and improve therapeutic outcomes in genetically diverse populations.

## Data Availability

The raw data supporting the conclusions of this article will be made available by the authors, without undue reservation.
