# Supplementary figures and images for: Impact of CYP3A5 1* and 3* single nucleotide variants on tacrolimus pharmacokinetics and graft rejection risk in pediatric kidney transplant patients
Source: Front Pharmacol. 2025 May 13;16:1592134. doi: 10.3389/fphar.2025.1592134 (PMC12107630; doi:10.3389/fphar.2025.1592134)

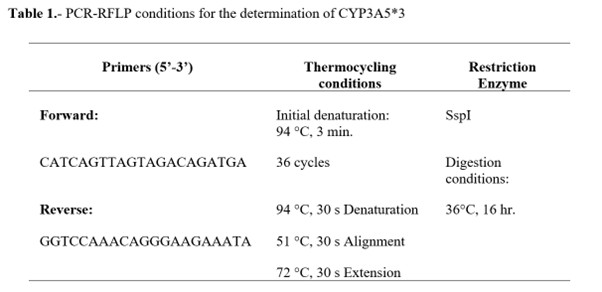

Supplement: Supplementary file 1 [file Image1.tiff]
